# Revisiting educational assumptions: The surprising negative link between creative extracurricular activities and creative thinking in PISA 2022

**DOI:** 10.1111/bjep.70066

**Published:** 2026-02-25

**Authors:** Sofiia Kagan, Denis Dumas, Yoojoong Kim

**Affiliations:** ^1^ Department of Educational Psychology University of Georgia Athens Georgia USA

**Keywords:** creative activities, creativity, divergent thinking assessment, PISA 2022, regression analysis

## Abstract

**Background:**

While skill development is generally linked to relevant practice, the 2022 PISA creative thinking report revealed a negative association between students' creative thinking performance and their engagement in creative activities.

**Aims:**

This study explored whether this negative association was linear, persisted across countries and remained after accounting for psychological and contextual moderators.

**Sample:**

We used PISA 2022 creative thinking assessment data. The final analytical sample included 372,193 students across 49 countries.

**Methods:**

We conducted correlation analysis and multiple regression with creative thinking as the outcome and creative activities as well as other contextual and psychosocial factors and their two‐way interactions with creative activities as predictors.

**Results:**

Creative thinking was negatively correlated with both in‐school (*r* = −.25) and out‐of‐school (*r* = −.32) activities, a pattern observed in 47 of 49 countries. After controlling for other predictors, both types of activities remained negative predictors of creative performance (*β* = −.06 and *β* = −.27, respectively). However, the interactions revealed that openness to intellect and creative self‐efficacy moderated the relation between out‐of‐school activities and creative thinking, showing small but significant positive associations, reversing the negative prediction. Additionally, feeling safe at school appeared to weakly but significantly moderate in‐school activities. It should be noted that the effect sizes were relatively small and accounted for a limited amount of variance.

**Conclusions:**

These findings suggest that creative activities may need restructuring to better support all students, and creativity measures likely need to continue to be refined.

A general theory to be seriously tested is that some primary abilities can be improved with practice of various kinds and that positive transfer effects will be evident in tasks depending upon those abilities. Guilford (1950, p. 449)

As pioneering psychologist of creativity J. P. Guilford conjectured in the above epigraph, it is often suggested in the literature that students' engagement in creative activities (e.g., putting on a play or designing a science experiment), might be expected to be positively associated with their creative thinking ability. This pattern could be observed because those activities support the development of creativity—the capacity to generate solutions that are not only useful or appropriate but also novel or original (Runco & Jaeger, 2012)—or it may be through a selection effect where more creative individuals choose to engage in such activities, or a combination of both (Carbonaro & Maloney, 2019; Diedrich et al., 2018; Dumas et al., 2021; Holland & Andre, 1987; Mack & Landau, 2015; Steger et al., 2022). In the educational context, such creative activities are often considered extracurricular, because traditional school curricula often emphasize solely convergent thinking, prompting students to engage with problems that have a single correct answer, as reflected in standardized testing practices (Beghetto & Kaufman, 2010; Beghetto & Plucker, 2006; Fuhrman, 2001; Robinson, 2006).

In a surprising twist, findings from the 2022 Programme for International Student Assessment (PISA, a large‐scale international assessment that evaluates the 21st‐century learning skills and literacies of 15‐year‐old students, including reading, mathematics and scientific thinking) challenged this assumption. The results revealed a negative association between participation in creative activities both inside and outside of school and performance on its creative thinking outcome measure (OECD, 2024a).

A potential misalignment between creative activities (which may primarily focus on artistic skills) and the creative thinking performance measure (which primarily assessed ‘students' capacity to think flexibly and make original and appropriate idea associations’ [OECD, 2024a, p. 196]) may help explain this counterintuitive association. Further analysis revealed that both extensive participation in activities and only sporadic participation were negatively associated with creative thinking performance, suggesting the existence of an optimal level of engagement in such activities, as highlighted in the PISA results report (OECD, 2024a). Barbot and Kaufman (2025) also pointed to the possibility of a nonlinear relation, while Kagan and Dumas (2025) highlighted potential methodological artefacts. For instance, the creative ability measure may have been overly focused on the utility of ideas, producing scores that may not have validly indicated creativity as a construct. In addition, the creative activities measure—which appeared vague and imprecise, not accounting for the breadth or intensity of activities—aggregated all domains into a single score, a choice that may not reflect the true multidimensionality of these activities. They further proposed some possible theoretical explanations for these counterintuitive findings, suggesting that creative activities might serve as a time to goof off, rather than as opportunities to develop creativity, thereby taking away crucial instructional time from core domains; or that gains in creativity during activities did not transfer to the assessment, or that highly creative adolescents might avoid adult‐led creative activities altogether.

While questions regarding the content and construct validity of PISA's creative thinking performance and PISA context questionnaire as well as potential methodological artefacts have been raised theoretically (Barbot & Kaufman, 2025; Goecke et al., 2025; Kagan & Dumas, 2025), the current empirical investigation focuses on elucidating this counterintuitive relation. Do creative activities genuinely enhance creative thinking, or does PISA offer a clue to a more complicated reality? The following sections critically examine existing research evidence regarding the relation between creative activity participation and measured creative thinking outcomes.

## The lack of support for creativity in the classroom

Creativity has been widely researched and recognized as a critical skill for more than a century (Galton, 1869; Guilford, 1950; Markey, 1935), but it has historically received limited attention in educational practice (Beghetto & Kaufman, 2014; Plucker et al., 2004; Sternberg & Lubart, 1998). However, recent developments underscore its renewed importance: the World Economic Forum (2015) identified creativity as one of the top skills essential for the future workforce, while the Organization for Economic Co‐operation and Development (OECD, 2019, 2024b) has formally incorporated creative thinking as an innovative domain in the PISA assessments of 2022. This inclusion reflects a growing consensus among policymakers that cultivating creativity at the national level is not merely beneficial, but imperative for future‐ready education systems (Zhao, 2015).

While fostering creativity in mainstream classroom settings poses significant pedagogical challenges (Beghetto, 2013), meta‐analytic evidence confirms that structured creativity training programs can effectively enhance creative competencies (Scott et al., 2004; Sio & Lortie‐Forgues, 2024; Valgeirsdottir & Onarheim, 2017). The primary difficulty stems from the need for teachers to reconcile their dual roles as facilitators of knowledge transmission on the one hand, and creativity cultivation, on the other hand, which often requires granting students greater autonomy that in turn may lead to disruption and challenges to classroom management (Beghetto, 2017; Davies et al., 2013). This pedagogical dilemma is further complicated by institutional expectations prioritizing rote or contrived outcomes over creative thinking (Kaufman & Sternberg, 2010; Runco et al., 2017).

Research shows that while teachers generally value creativity and believe in their students' creative potential (Kampylis et al., 2008; Runco & Johnson, 2002), a persistent gap exists between these positive attitudes and actual classroom practices (Aljughaiman & Mowrer‐Reynolds, 2005; Cropley, 2010; Kagan et al., 2025; Mullet et al., 2016; Scherbakova et al., 2024; Westby & Dawson, 1995). This implementation gap stems partly from inadequate teacher training, as preservice programs rarely include comprehensive instruction on teaching for creativity (Anderson et al., 2021; De Souza Fleith, 2000). When such training does exist, it typically falls under accelerated, advanced, or gifted education programs, leaving general classroom instruction largely devoid of systematic creativity development (Besançon, 2013; Collard & Looney, 2014).

## Extracurricular activities as a support for creativity

Despite the limited emphasis on creativity in standard curricula, schools and other educational institutions usually believe they can foster creative development through structured extracurricular opportunities—both school‐based (e.g., after‐school computer programming classes) and community‐based (e.g., private dance academies). These activities, ranging from creative writing workshops to music classes and Science, Technology, Engineering and Mathematics (STEM) programs, share three key characteristics empirically linked to creative growth: (1) increased student autonomy, (2) structured opportunities for choice, imagination and exploration and (3) encouraging students' intrinsic motivation (Beghetto & Kaufman, 2014, 2010). Such environments align with classic principles of creative development from educational psychology, such as those forwarded by Vygotsky (1993/2004), where guided freedom within domain‐specific constraints optimally fosters innovation.

It is widely empirically supported that engagement in creative activities fosters creativity development (e.g., Diedrich et al., 2018; Silvia et al., 2012). Self‐reported creative activity participation has been shown to serve as a valid proxy for creativity measures, showing consistent positive correlations with established assessments (Dollinger et al., 2004; Dumas et al., 2021; Furnham et al., 2008; Steger et al., 2022). Beyond enhancing creativity‐specific outcomes, extracurricular activities are associated with broader benefits, including academic achievement benefits and psychological well‐being (Feldman & Matjasko, 2005; Mahoney et al., 2003), and the development of personality and motivational characteristics such as openness to experience and intellect and self‐efficacy (Holland & Andre, 1987; Lee & Kemple, 2014; Tan et al., 2016); characteristics that are themselves established predictors of creativity (Beghetto, 2006; Dollinger et al., 2004; McCrae, 1987; Tierney & Farmer, 2011).

However, the relation between extracurricular activities and student outcomes is neither uniformly linear nor consistent. For creativity‐related outcomes, research demonstrates significant variation based on activity type, intensity and the measures used (Diedrich et al., 2018). For instance, Cotter et al. (2015) found that participation in sports (which might appear to provide more opportunities for creative thinking than in‐school curricula) was negatively associated with creativity, while intensive involvement in academic activities showed diminishing returns in divergent thinking and other measures of creativity. Similarly, Cortez and Montes (2025) reported only moderate correlations between arts‐based activities and creativity, with no significant relation to time invested.

Earlier work by Milgram and Milgram (1976) found that creative thinking performance on the Wallach and Kogan Creativity Battery (consisting of alternate uses, pattern meanings, similarities and line meanings tasks) correlated positively with participation in creative activities such as science, social leadership, creative writing and fine arts, but not with drama or music. Moreover, the correlations were different by gender: for female students, creative thinking scores were significantly and positively associated only with participation in fine arts activities (*r* = .26) and writing (*r* = .23), whereas for male students, creative thinking scores correlated positively with social leadership (*r* = .40), writing (*r* = .31), community service and fine arts (*r* = .21; Milgram & Milgram, 1976). In one of the recent studies, Cortez and Montes (2025) examined extracurricular participation in artistic activity (dance, music, drawing and painting, literature and poetry) and its influence on divergent thinking performance. Using the Divergent Association Task, in which participants were asked to generate 10 words as different from each other as possible, they found that students who participated in at least one activity scored on average two points higher on the divergent thinking test (*M* = 78.38, *SD* = 5.86) than those who did not participate in any activities (*M* = 76.39, *SD* = 4.74); the mean difference between groups: *t*(40) = −1.69, *p* = .049. This *p*‐value was just at the threshold of statistical significance, and the effect size we calculated based on the reported statistics would be considered moderate in educational research (Cohen's *d* = .37). However, the amount of participation did not significantly influence creativity. While the authors argue for the importance of extracurricular participation, their findings need replication and further scrutiny in disentangling the effects of specific creative activities on creative performance.

For academic outcomes, studies reveal equally complex patterns. While most of them demonstrated positive associations, the magnitude of effects varied significantly across educational contexts (Dang, 2022; Feldman & Matjasko, 2005; Kravchenko & Nygård, 2022; Mahoney et al., 2005; Marsh, 1992; Vandell et al., 2015), with evidence of curvilinear relations (Fredricks, 2011; Marsh & Kleitman, 2002). Some research showed that extracurricular activities had no significant effects on academic outcomes (e.g., Tompsett & Knoester, 2023).

Moreover, the causal mechanisms through which extracurricular activities may enhance student outcomes remain unclear. Some studies suggested these benefits operate through the development of non‐cognitive skills (e.g., interpersonal competencies; Covay & Carbonaro, 2010; Eccles et al., 2003) or through social capital gains from peer networks established via participation (Gibbs et al., 2014; King et al., 2020). Research also examines socioeconomic status (SES) and race as potential mediators of this relation (Covay & Carbonaro, 2010).

These findings collectively indicate that the benefits of creative activities may depend critically on the quality and quantity of participation, the specific type of activity and the targeted outcomes being measured.

## PISA 2022 assessed creative thinking and participation in extracurricular activities

Traditional educational research, from which the findings above originate, predominantly focuses on assessing student‐level outcomes. A central aim of this research lies in examining students' cognitive and affective attributes, with the dual objective of deriving meaningful inferences about individual learners and identifying pedagogical strategies to foster these competencies (Alexander, 2013; Dumas & Edelsbrunner, 2023; Thurstone, 1926).

Unlike small‐scale assessments, PISA employs a large‐scale design to generate population‐level inferences (OECD, 2019, 2024a). The 2022 cycle marked a significant advancement by introducing creative thinking as an innovative domain of assessment, representing the first international large‐scale measurement of this construct. Notably, PISA also collected data on students' participation in creative extracurricular activities both inside and outside of school, revealing a counterintuitive negative association between activity engagement and creative thinking performance. It should be noted that all questionnaire responses across different activities (e.g., art classes, creative writing, debate club) were aggregated into two separate overall scores: one for creative activities inside school and one for activities outside school (OECD, 2024b). Each score is an Item Response Theory (IRT) scaling of students' reported frequency of participation in a range of specific activities.

As the most comprehensive international educational dataset available, PISA provides an unparalleled opportunity to rigorously examine this paradoxical relation through secondary data analysis.

While PISA data may offer valuable macro‐level insights into variable distributions (Hopfenbeck et al., 2018), its analytical potential is constrained by the conditioning model used to generate the test scores for performance assessment (termed plausible values; Marsman et al., 2016) for each student. This model incorporated background questionnaire variables, which likely included students' participation in creative activities inside and outside of school, to impute creative thinking scores (OECD, 2024b). This issue limits secondary analysts to examining mostly relations that were included in that conditioning model rather than discovering novel pathways (Dumas et al., 2026; Rutkowski & Rutkowski, 2016). Thus, analysing large‐scale data requires specific methodological considerations (Emler et al., 2019). For instance, researchers must work with plausible values—multiple model estimates of students' proficiency drawn from a posterior distribution of latent ability—rather than individual point estimates of latent scores (Mislevy et al., 1992; von Davier et al., 2009; Wu, 2005). Additionally, PISA's complex sampling design requires the use of replicate weights to account for planned missingness and ensure accurate variance estimation, as no student completes the full assessment battery or even an entire domain's items (OECD, 2024b; Rutkowski et al., 2010). In fact, most students in the PISA 2022 publicly available dataset never encountered the creative thinking measure at all, yet their model‐based proficiency estimates are still represented in the dataset through plausible values. Please note that the creative thinking scores in PISA are plausible values, but the questionnaire scores are not; they are IRT‐scaled point estimates. These methodological nuances will be discussed in detail in the subsequent methodology section.

## Testing the assumptions: Goals of present study

The present study examines whether the negative association between in‐school and out‐of‐school extracurricular creative activities and creative thinking performance reported by OECD (2024a) remains consistent across all participating countries and after controlling for relevant contextual and psychoeducational covariates. These include openness to intellect, creative self‐efficacy, psychological safety at school, SES, gender and immigrant background. Furthermore, we investigate potential moderation effects of these variables on the relation between creative activities and creative thinking performance. The proposed variables seem to be logical moderators between participation in creative activities and creative thinking, both based on the PISA results report and prior research on creativity. According to the PISA results report, ‘weekly participation in school activities is positively associated with a range of student attitudes that in turn support creative thinking, such as creative self‐efficacy and openness to intellect’ (OECD, 2024a, p. 197), which emphasized the potential moderating role of these variables in the relation between creative activity participation and creative thinking performance. Moreover, prior research consistently highlighted that a supportive creative environment was of paramount importance for fostering students' creativity (Beghetto & Kaufman, 2014; Craft, 2005). A fundamental component of such an environment is that students feel both psychologically and physically safe at school. This sense of safety provided the foundation for engagement and exploration, making the environment conducive to creativity development (Kutsyuruba et al., 2015). When students' basic psychological and biological needs (including the need for safety) are not met, it may be unreasonable to expect them to be productive in terms of learning new skills or engaging in creative processes (Amabile et al., 1996; Gietz & McIntosh, 2014; Maslow, 1943).

Demographic variables such as SES, gender and immigrant background should also be included in the analysis, as they are well‐documented predictors of student outcomes and may account for part of the observed relations between creative activities and creative thinking in educational research (Auwarter & Aruguete, 2008; Brandmiller et al., 2020). By including these demographic factors, the analysis reduces the risk of attributing effects on creative thinking to activity participation when they may instead reflect underlying demographic disparities.

Descriptive analyses examining this empirical pattern of the relation between creative activities participation and creative thinking performance are included both in the OECD (2024a) report for the PISA creative thinking assessment, as well as in Hernández‐Ramos and Araya (2025), but a full analysis of the issue with appropriate methodological steps needed to recover the population patterns from the PISA conditioning model (i.e., representative weighting, aggregation of findings across plausible values) has not yet been attempted.

## METHODOLOGY

### Dataset

The analysis was conducted using data from the Programme for International Student Assessment (PISA; https://www.oecd.org/pisa/data/2022database/).

### Participants

In 2022, approximately 690,000 students participated in the PISA assessment, representing a population of around 29 million 15‐year‐olds enrolled in schools across 81 countries and economies (OECD, 2024b). PISA students were between 15 years and 3 months and 16 years and 2 months old at the time of assessment, having completed at least 6 years of formal schooling. As part of the assessment design, about 28% of the sampled students were assigned the creative thinking assessment. After excluding countries that did not meet OECD sampling guidelines for result interpretation (i.e., school‐level exclusions and within‐school exclusions combined do not exceed 5%; the final weighted school response rate is at least 85% of sampled schools; the student response rate is at least 80% of all sampled students across responding schools; OECD, 2024b) and those countries without data relevant to our analysis, the final analytic dataset used here included 372,193 students (185,800 females, 49.9%), with a mean age of 15.8 years (Median = 15.75, *SD* = 0.291). The excluded countries were Israel, Spain, Denmark, Costa Rica, Germany, Republic of Moldova, Austria, Australia, Canada, Hong Kong, Jamaica, Latvia, the Netherlands, New Zealand and Panama. Please refer to Appendix A in the supplementary materials for the total number of students included in the creative thinking performance measure, broken down by country. A total of 49 countries were included in the analysis, with sample sizes ranging from 3127 students in Malta to 24,600 students in the United Arab Emirates.

### Measures

All the measures were developed and administered by PISA; see the relevant reports for further reference (OECD, 2024a, 2024b). It should be noted that because the scores for performance measure are plausible values featured in a publicly available dataset, and the self‐reported assessments are IRT‐scaled indices (with most students completing the background questionnaires fully; see OECD, 2024b, p. 93, for more information on the sampling design), we do not report reliability for the measures in the conventional way. Reliabilities of the measures are reported in the PISA 2022 technical report (OECD, 2024b). For the creative thinking assessment, reliability was estimated at .80 (OECD, 2024b, p. 300). For the contextual self‐report questionnaires, reliability coefficients were provided separately for each country, with most exceeding .80 (OECD, 2024b, p. 443).

#### Creative thinking

PISA developed an assessment to measure students' creative thinking across four domains: social and scientific problem‐solving, visual expression and written expression. The test targeted three core ideation processes: generating diverse ideas, generating creative ideas and evaluating and improving ideas. The PISA technical report specified that the creative thinking assessment consisted of 21 units (i.e., conceptually grouped clusters of test items) in total. ‘Each unit varied according to the ideation process involved, the unit length, the number of items in the unit, and the domain context’ (OECD, 2024a, p. 48). The units were organized into five mutually exclusive 30‐min blocks. The blocks were rotated according to a balanced rotation design in which different groups of students were randomly assigned to different test blocks, ensuring that each unit was administered to a representative sample of students while keeping individual testing time limited (OECD, 2024b). For examples of items, see the PISA results report (2024a, pp. 53–68); a few examples are provided below.

Each unit included two out of the three ideation processes: generate diverse ideas, generate creative ideas and evaluate and improve ideas. For example, unit 1 in the visual domain included items that measured generating creative ideas as well as evaluating and improving ideas, while unit 2 in the visual domain included items measuring the generation of diverse ideas and evaluation and improvement of ideas (OECD, 2024b). The PISA results and technical reports do not specify the number of items in each unit; instead, they provide only examples of items for each domain, covering nine units in total that measured different ideation processes.

In the written expression domain, the ‘Space Comic’ item, measuring the process of generating creative ideas, asked students to write an original dialogue between the Sun and the Earth. To assess generating diverse ideas, the ‘Illustration Titles’ item asked students to write three different titles for an abstract illustration of an oversized book embedded in nature.

In the visual expression domain, one task measuring generation of creative ideas required students to design a poster for a science fair themed ‘Life in Deep Space’. A second item in the same unit, measuring evaluation and improvement of ideas, provided students with a simple poster design (the Sun and one planet) and asked them to improve it by connecting it to the topic of ‘Life in Deep Space’ in an original way.

In the social problem‐solving domain, the ‘Save the Bees’ task, measuring generation of creative ideas, asked students to suggest three different ideas for raising awareness about the importance of bees. The ‘Library Access’ task, measuring generation of diverse ideas, required students to propose three different ideas for improving wheelchair accessibility in a library.

In the scientific problem‐solving domain, the ‘Save the River’ unit asked students in a generating diverse ideas task to propose two original causes of reduced frog populations other than river pollution. In a subsequent item measuring evaluation and improvements of ideas, students were asked to suggest original ways to improve an experiment on water pollution.

The responses were scored based on the degree of originality and appropriateness. To earn full credit, responses had to demonstrate originality. Responses that reflected conventional themes and were appropriate, but not original, were awarded partial credit (conventional themes were given in the scoring manual; [OECD, 2024a, pp. 53–68]).

Plausible values for creative thinking (estimates of students' proficiency) were imputed using a latent regression model that incorporated information from students' background questionnaires and their performance in other academic domains, such as math and reading. Instead of providing a single point estimate, this model generated several plausible values to account for uncertainty in measuring students' abilities (OECD, 2024b).

#### Creative activities inside and outside of school

Creative activities were measured using a self‐report Likert‐scale, in which students indicated how often they participated in various activities (art classes/activities, creative writing classes/activities, music classes/activities, debate club, dramatics, theatre classes/activities, publications, science club, computer programming classes/activities) both inside and outside of school (see Table 1). The response options were: ‘Never or almost never’, ‘About once or twice a year’, ‘About once or twice a month’, ‘About once or twice a week’ and ‘Every day or almost every day’. Please refer to Appendix B and Appendix C in the supplementary materials for the number of students with available data on creative activities inside and outside of school, respectively, broken down by country. It should be noted that the OECD used IRT to scale the indices, resulting in two scores: one for activities inside of school and one for activities outside of school. We examined skewness and kurtosis for each of the continuous variables (Table 2), and both creative activities inside and outside of school fell within the commonly acceptable ranges (Curran et al., 1996; Hair et al., 2010). However, when inspecting the histograms, we observed that despite the IRT normality assumption and acceptable skewness and kurtosis values, the distribution of activities inside of school appeared somewhat bimodal, while activities outside of school appeared somewhat zero‐inflated, with the mode at the lowest scale value. We acknowledge this in the discussion session as a potential limitation of the study.

**TABLE 1:** Items for creative activities inside and outside school.

1	Art classes/activities (e.g., painting, drawing)
2	Creative writing classes/activities
3	Music classes/activities (e.g., chorus, band)
4	Debate club
5	Dramatics, theatre class/activities
6	Publications (e.g., newspaper, yearbooks, literary magazine)
7	Science club
8	Computer programming classes/activities

*Note*: The prompt for creative activities inside of school was: ‘Indicate how often you participate in each activity listed below in your school. Indicate if it is not available at your school’. The prompt for creative activities outside of school was: ‘Indicate how often you participate in each activity listed below outside of school. Indicate if it is not available near your home’.

**TABLE 2:** Skewness and kurtosis values for continuous variables.

Variables	Skewness	Kurtosis
Activities inside school	1.10	2.56
Activities outside school	1.13	1.53
Creative self‐efficacy	.11	.63
Openness to Intellect	.36	.89
Feeling safe	−.18	−1.00
SES	−.56	.10

#### Creative self‐efficacy

The index of creative self‐efficacy was measured using 10 items in which students rated their confidence in performing creative tasks (e.g., ‘Coming up with creative ideas for school projects’, ‘Inventing new things’.) Responses were given on a four‐point scale: ‘Not at all confident’, ‘Not very confident’, ‘Confident’ and ‘Very confident’.

#### Openness to intellect

The index of Creativity and Openness to Intellect was measured using 10 items in which students rated their agreement with statements about their openness to intellect (e.g., ‘Doing something creative satisfies me’, ‘I like games that challenge my creativity’.) Responses were recorded on a four‐point scale: ‘Strongly disagree’, ‘Disagree’, ‘Agree’ and ‘Strongly agree’.

#### Feeling safe

Students rated their agreement with four statements about their perceived safety (e.g., ‘I feel safe on my way to school’, ‘I feel safe in my classrooms at school’.) These responses were combined into the Feeling Safe index. Each statement was rated on a four‐point scale: ‘Strongly agree’, ‘Agree’, ‘Disagree’ and ‘Strongly disagree’.

#### Socioeconomic status (SES)

In the PISA technical report, SES is referred to as the Index of Economic, Social and Cultural Status (ESCS). This is a composite index based on three components: the highest parental occupation status, the highest parental education (in years) and household possessions. For clarity, we refer to this construct simply as SES throughout the paper.

#### Categorical variables

##### Gender

Student gender was obtained from school records in the sampling data and validated against students' questionnaire responses, with females set as the reference group.

##### Immigrant Background

The index of immigrant background consists of three categories: (1) native students, defined as those with at least one parent born in the country/economy of assessment; (2) second‐generation students, defined as those born in the country/economy of assessment whose parents were born abroad; and (3) first‐generation students, defined as those born outside the country/economy of assessment whose parents were also born abroad. We recoded these three groups into a dichotomous variable by combining the first two categories (native and second‐generation students) as the reference group to indicate whether the child themselves had immigrated. The recoding was intended to facilitate clearer interpretation and a more straightforward analysis.

### Data analysis overview

#### Open access code for replication and extension

We have made the full R code used for this analysis, along with the cleaned data file and data selection procedures, publicly available on OSF to ensure transparency and facilitate easy replication: https://osf.io/txagq/overview?view_only=0a6b5fc06fa449fcb285b55f520f67c5.

#### Aggregating findings across plausible values

PISA does not report single test scores for each student but instead provides multiple plausible values—random draws from a posterior distribution of latent proficiency estimates (OECD, 2024b). These values are not individual‐level test scores, but rather psychometrically derived estimates that allow for unbiased population‐level inferences (Mislevy et al., 1992; von Davier et al., 2009).

Given this design, using only one plausible value per participant can lead to underestimation of variance and biased results. To account for this, we included all 10 plausible values provided for the creative thinking outcome in analyses. For both correlation and regression modelling, we conducted separate analyses for each plausible value and then combined the results using Rubin's rules (Rubin, 1987). In practice, this meant estimating the correlations and regression coefficients 10 times, once for each plausible value and then pooling the results. Rubin's rules combine the estimates by taking the average of the coefficients across all imputations as the final point estimate, while the variance is computed by incorporating both the *within‐imputation variance* (the average of the variances from each analysis) and the *between‐imputation variance* (the variability of the estimates across imputations). This approach allows for correct estimation of parameter means and associated variances by accounting for both the within‐ and between‐imputation variability, thus ensuring valid statistical inference.

Although plausible values are not suitable for individual‐level predictions, they are an established and robust method for estimating population characteristics and examining relations among variables (Marsman et al., 2016; Wu, 2005). This method respects the imputation‐based nature of the proficiency scores and allows for reliable estimates of associations between creative thinking and its predictors.

#### Weighting participant scores based on sampling design

To account for the complex survey design used in PISA, including its planned missingness structure and multi‐stage stratified sampling, we applied both whole sample weight and 80 replicate weights in our analyses. The whole sample weight (also called the student weight) ‘ensures that each participating student appropriately represents the correct number of students in the full PISA population’, enabling population‐weighted parameter estimates (OECD, 2024b, p. 188). The 80 replicate weights were used to estimate sampling variance, accounting for nonresponse, planned missingness and the stratified and clustered nature of the PISA sample design. Applying both types of weights is essential for producing unbiased estimates and accurate standard error estimates (Rust & Rao, 1996). We used the weights package in R (Pasek, 2018) to compute weighted correlations, and the survey package in R (Lumley, 2004) to incorporate the full complexity of the PISA design into regression analyses.

#### Running correlations and regression models

The analysis proceeded in two main steps: (1) we conducted weighted Pearson correlations between all the variables of our interest and (2) we conducted regression analyses to predict creative thinking performance using the following predictors: participation in creative activities (inside and outside of school), openness to intellect, creative self‐efficacy, psychological safety at school and demographic variables (gender, immigrant status and SES). The regression models included all two‐way interaction terms between participation in creative activities (both inside and outside of school) and the other predictor variables. All the continuous variables, including the plausible values, were standardized prior to analyses for ease of interpretation.

#### Correcting significance tests based on country‐level clustering

To address the nested structure of the data at the country level, all regression models employed a multilevel standard error correction method (Bauer et al., 2020) to ensure the accuracy of statistical inferences. Robust standard errors were computed using the sandwich package in R (Zeileis, 2004; Zeileis et al., 2020), separately for each plausible value, and then combined using Rubin's rules (Rubin, 1987). A full multilevel regression analysis allowing for country specific slopes for each predictor was not conducted, as preliminary analyses with a two‐level random‐intercept model (i.e., with no predictors other than the country code) indicated that the country‐level random intercept was not statistically significant and offered little improvement over the single‐level model. To assess the proportion of variance in creativity scores attributable to country‐level differences, we calculated the intraclass correlation coefficient (ICC) from these two‐level random‐intercept models for each plausible value. ICC represents the proportion of total variance explained by between‐country variation relative to total variance (between‐ plus within‐country). The average ICC across plausible values was approximately .25, indicating that about 25% of the variance in students' creativity scores is due to differences between countries, whereas the remaining 75% reflects differences between students within countries. All psychological predictors showed low ICCs (ranging from .029 to .037). Creative activities inside school had an ICC of .111 and outside school had an ICC of .135. Gender showed negligible variance at the country level (ICC = .001). However, immigrant background and SES demonstrated higher ICCs (.423 and .214, respectively), which is expected given inter‐country variation in immigration rates and economic levels. ICCs were calculated using the lme4 package (Bates et al., 2015).

As all variables of interest demonstrated low to moderate ICCs (except for immigrant background), and estimating random effects for 49 countries with 20 predictors (including two‐way interactions) would be computationally infeasible, the use of robust standard errors is therefore a sufficient and pragmatic approach to account for the data's hierarchical structure (Hedges & Hedberg, 2007; McNeish, 2014).

## RESULTS

### Pearson bivariate correlations

#### Overall correlations

All correlations between the variables of interest are reported in Table 3. The correlation with creative thinking performance was significantly negative for both types of activities (*r* = −.25 for inside school; *r* = −.32 for outside school). Creative activities also significantly and negatively correlated with SES (*r* = −.10 for inside school; *r* = −.14 for outside school). Creative thinking showed the strongest significant positive correlation with SES (*r* = .41). Other variables appeared to have only weak associations with creative thinking ability.

**TABLE 3:** Pearson bivariate correlations.

	Activities inside school	Activities outside school	Self‐efficacy	Openness	Feeling safe	Creative thinking	SES
Activities inside school	1						
Activities outside school	.78	1					
Self‐efficacy	.18	.17	1				
Openness	.15	.16	.50	1			
Feeling safe	.02	.01	.12	.11	1		
Creative thinking	−.25	−.32	.11	.12	.06	1	
SES	−.10	−.14	.13	.09	.10	.41	1

*Note*: All correlations are statistically significant at the *p*‐value level of < .001. Rubin's rules were used to combine the estimates across the 10 plausible values, accounting for both the within‐ and between‐imputation variability.

#### Country‐level correlations between creative thinking and creative activities

The correlations between creative activities and creative thinking performance by country followed a consistent pattern, with most showing negative relations. These negative correlations ranged from −.03 (Chinese Taipei) to −.27 (Slovak Republic) for creative activities inside school (all of them were statistically significant at this sample size). The only exceptions were Korea and Macao, which showed slightly positive correlations of .09 and .07, respectively. These findings might suggest that Korea and Macao seem to be leveraging creative activities, at least inside of school, more effectively than other nations.

For creative activities outside of school, all correlations were significant and negative, ranging from −.06 (Singapore) to −.31 (Slovak Republic). The correlation for Korea was the only value not significantly lower than zero (i.e., *r =* −.01).

### Multiple regression analysis

#### Comparing linear and nonlinear relations

Before conducting the full linear regression analysis with all variables of interest, we first examined the form of the relation between creative activities and creative thinking performance in two sets of models, analysed separately for activities inside and outside of school. For each, we ran two models: one with only the linear term (e.g., creative activities inside school predicting creative thinking performance) and another that included both the linear and quadratic terms. All models accounted for replicate and survey weights and used all 10 plausible values of the creative thinking performance outcome.

Although the quadratic terms for creative activities both inside and outside of school were statistically significant (outside: *β* = .063, *SE* = .0025, *p* < .001; inside: *β* = .027, *SE* = .0015, *p* < .001), their contribution to model fit was minimal. Specifically, adding the quadratic term improved *R* ^2^ by approximately .002 across plausible values for outside‐of‐school activities and by less than .001 for inside‐of‐school activities, reflecting a negligible increase in explained variance. Therefore, we decided not to include the quadratic term in the final regression model and focus only on a linear relation between creative activities (outside and inside of school) and creative thinking performance.

#### Main effects from linear model

The full linear regression model predicting creative thinking included the following independent variables: creative activities inside and outside of school, openness to intellect, psychological safety at school, creative self‐efficacy, immigrant background, gender and SES, along with interaction terms between creative activities inside and outside of school and all the other variables of interest. The results, including beta coefficients, robust standard errors (*SE*), robust *p*‐values and effect sizes (partial eta squared), are reported in Table 4. The model had a pseudo‐*R* ^2^ = .54, which reflects the improvement of the fitted model over a null (intercept‐only) model under the survey‐weighted framework. It should be noted that this pseudo‐*R* ^2^ is based on model deviance and therefore differs from the classical variance‐based *R* ^2^: it indicates relative model fit rather than the exact proportion of variance explained in the outcome (Lumley, 2017). We also calculated a more traditional variance‐based *R*‐square, which equalled .27.

**TABLE 4:** Standardized regression coefficients predicting creative thinking scores.

Term	*β*	*SE*	*p*	Robust *SE*	Robust *p*	*η*_p_^2^
(Intercept)	.01	.01	.211	.007	.160	<.001
Activities outside school	−.27	.01	<.001	.006	<.001	.006
Activities inside school	−.06	.01	<.001	.005	<.001	<.001
SES	.35	.01	<.001	.004	<.001	.023
Gender (male)	−.14	.01	<.001	.008	<.001	<.001
Immigrant background	−.01	.02	.664	.009	.439	<.001
Self‐efficacy	.06	.01	<.001	.004	<.001	.001
Openness	.10	.01	<.001	.004	<.001	.002
Feeling safe	.02	.004	<.001	.003	<.001	<.001
Activities inside × SES	−.002	.003	.525	.002	.210	<.001
Activities inside × Gender (male)	.02	.01	.010	.007	<.001	<.001
Activities inside × Immigrant	−.02	.02	.462	.017	.350	<.001
Activities inside × Self‐efficacy	−.03	.01	<.001	.003	<.001	<.001
Activities inside × Openness	−.01	.01	.011	.003	<.001	<.001
Activities inside × Feeling safe	.01	.004	.001	.003	<.001	<.001
Activities outside × SES	−.06	.01	<.001	.003	<.001	.001
Activities outside × Gender (male)	−.04	.02	<.001	.007	<.001	<.001
Activities outside × Immigrant	−.02	.004	.193	.001	.005	<.001
Activities outside × Self‐efficacy	.04	.01	<.001	.008	<.001	.001
Activities outside × Openness	.03	.01	<.001	.003	<.001	<.001
Activities outside × Feeling safe	−.003	.01	.568	.003	.424	<.001

*Note*: Deviance‐based *R* ^2^ = 0.54. Variance‐based *R* ^2^ = 0.27. Reference group for gender is female. Reference group for immigrant background is defined as those born in the country of assessment (native or second‐generation students). The total analytical sample size, after listwise deletion, was 230,951 students. Rubin's rules were used to combine the estimates across the 10 plausible values, accounting for both the within‐ and between‐imputation variability.

We found that both creative activities inside and outside of school significantly and negatively predicted creative thinking performance (*β* = −.06 and *β* = −.27, respectively). Notably, after controlling for all other variables in the model, out‐of‐school creative activities had a stronger negative association with creative performance than in‐school activities. SES positively predicted creative thinking performance (*β* = .35), while being male was associated with lower creative performance (*β* = −.14). Immigrant background—defined as being born outside the country of assessment with both parents also born in another country—was slightly negatively but not significantly associated with creative thinking performance (*β* = −.01, *p* = .664), and the effect was very close to zero. Additionally, both creative self‐efficacy and openness to intellect were weak but positive predictors (*β* = .06 and *β* = .10, respectively). Feeling safe at school also positively predicted creative thinking performance, though the effect was close to zero (*β* = .02). While most robust *p*‐values were statistically significant, the large sample size diminishes their practical interpretability. Therefore, we also examined effect sizes using partial eta squared (Lakens, 2013; Muller & Cohen, 1989), which are available in Table 4.

#### Interaction effects

We observed several notable and statistically significant interaction effects. Creative activities inside of school interacted with students' sense of safety at school, positively predicting creative thinking (*β* = .01). A similar trend was observed for gender with a small positive effect (*β* = .02). While females showed an overall advantage in creative thinking (main effect), males who participated in in‐school creative activities scored slightly higher. Creative self‐efficacy and openness to intellect moderated the negative relation between inside‐school creative activities and creative thinking. These interactions negatively predicted creative thinking ability, but the strength of these associations was weaker than the main effect of creative activities inside of school (*β* = −.03, *β* = −.01, respectively).

For creative activities outside of school, the interactions with creative self‐efficacy and openness to intellect positively predicted creative thinking performance (*β* = .04; *β* = .03), moderating the negative relation between creative activities and creative thinking performance. Interactions between creative activities outside of school and SES, immigrant background and gender negatively predicted creative thinking, but the strength of these associations was weaker than that of the main effect (*β* = −.06, *β* = −.02 and *β* = −.04, respectively).

## DISCUSSION

The consistent negative correlations observed across most countries, combined with the regression results showing significant negative predictions of creative thinking ability by engagement in creative activities both inside and outside of school, highlight a counterintuitive pattern that warrants serious attention. Some measurement artifactual limitations of the OECD endeavour should be acknowledged. These include the convergent nature of scoring and task structure in the creative thinking assessment, the aggregation of scores from different dimensions and ideation processes into a single composite score, and the vagueness of the creative activity questionnaire (which does not adequately capture the quality or intensity of activities, nor provide separate scores for each activity type). In addition, the large‐scale assessment design relied on planned missingness and plausible values rather than estimated scores for respondents (Dumas et al., 2026; Kagan & Dumas, 2025). Despite these limitations, our findings align with prior research showing complex relations between extracurricular activity participation and student outcomes (Marsh, 1992; Marsh & Kleitman, 2002). This underscores the importance of examining not only the quantity of participation but also the quality of activities and their differential impacts on specific students' outcomes, while also considering the nature of the creative thinking assessment and its potential influence on the observed relation.

For example, Cotter et al. (2015) demonstrated that the correlation between extracurricular participation and creativity depends on the type of activity, the intensity of participation and the creativity measure employed. Specifically, they reported that membership in art clubs did not predict self‐reported artistic creativity but did predict self‐reported performance creativity within the domain of performance as measured by the Kaufman Domains of Creativity Scale (K‐DOCS; Kaufman, 2012). Moreover, participation in athletic clubs was unrelated to creativity measures, further emphasizing the importance of distinguishing among activity types and recognizing the role of measurement approaches in shaping observed associations (Cotter et al., 2015). Farb and Matjasko (2011) argued that a broader perspective should be adopted when considering the influence of extracurricular activity participation on adolescent developmental outcomes. Such an approach should take into account the intensity, breadth and duration of participation, as well as characteristics of the activities themselves (e.g., adult supervision).

The stronger negative association with creative activities outside of school may suggest that greater involvement in extracurricular activities reduces time available for formal academic instruction. This trade‐off could lead to lower performance on PISA creative thinking assessment, as PISA operationalized creative thinking primarily as a convergent process, emphasizing the usefulness or appropriateness of responses over their originality or novelty, and also constraining the number of responses to one to three ideas (Acar & Shen, 2025; OECD, 2024a). Indeed, the creative thinking scores were found to correlate strongly with reading and math achievement, further supporting this interpretation (*r* = .66; *r* = .67; OECD, 2024a). This operationalization may partly explain the observed negative associations: strong involvement in open‐ended, less structured creative activities may have developed divergent thinking skills that were not well captured by the PISA assessment format. Consequently, students engaged in creative pursuits may have been disadvantaged by the test's convergent orientation. This mirrors findings by Cotter et al. (2015), who observed a negative relation between intensive academic‐related activity participation and performance on creativity tasks, suggesting a bidirectional effect: intensive focus on either academic or creative pursuits may come at the expense of the other domain's assessed competencies. If there is a misalignment between the two, extracurricular creative activities emphasize divergent thinking, autonomy and free exploration, while the PISA creative thinking test requires scientific knowledge and convergent thinking and vice versa, the relation between the two may be negative. South Korea, for example, one of the highest‐performing countries in reading, math and science skills (OECD, 2024a), exhibited a different pattern, with a low trade‐off between the two, as supported by our correlation findings (the only country for which the correlation between in‐school creative activities and creative thinking performance was positive). Korea may experience less of this trade‐off between creative activity participation and domain‐specific knowledge, suggesting that extracurricular activities in the Korean context—where academic performance is highly valued—may place stronger emphasis on academic advancement.

Another possible explanation emerges from the distribution of activity scores. The histogram of creative activities outside of school revealed a possible zero‐inflated pattern, with the mode at the lowest score, indicating that zero activities may have been the most common response to the scale. A likely explanation is the cost barrier for outside of school activities. In contrast, activities inside of school exhibited a somewhat bimodal distribution, suggesting broader access, likely because schools often provide such activities for free. This pattern highlights the importance of school‐based creative opportunities while also pointing to a potential statistical issue: zero‐inflation in the outside of school activity measure could potentially influence analyses, contributing to the observed negative association.

Importantly, interaction results add nuance to this picture. Creative activities outside of school positively predicted creative thinking performance when moderated by openness to intellect or creative self‐efficacy. This finding aligns with the PISA results report, which highlighted that students' capacity to generate diverse and original ideas is associated with personal attributes such as openness to intellect and creative self‐efficacy (OECD, 2024a). Openness to experience and intellect are among the most strongly associated personality traits with creativity performance measures as well as with self‐reported measures of creative activities, achievements and behaviours (Diedrich et al., 2018; Kaufman, 2012). Individuals high on openness may tend to engage more in creative behaviours and exhibit greater cognitive flexibility (Chen et al., 2019), which are essential for creative thinking. Similarly, creative self‐efficacy has been shown to positively influence creativity (Tierney & Farmer, 2011). The observed relations may stem from self‐selection, where individuals with high self‐efficacy and traits such as openness are more likely to participate in creative activities. Alternatively, engaging in creative activities may enhance these qualities, thereby fostering creative thinking abilities, as suggested by the PISA results report (OECD, 2024a).

Similarly, feeling safe at school appeared crucial for school‐based activities to predict higher creative thinking scores. This finding aligns with previous research emphasizing the importance of a safe and supportive environment in fostering creativity. In educational settings, psychological safety has been linked to enhanced academic performance across domains (Gietz & McIntosh, 2014; Kutsyuruba et al., 2015). Extending this to organizational settings, Yaqoob et al. (2024) found that psychological safety significantly predicted creativity, accounting for 47% of its variance by encouraging risk‐taking and idea‐sharing. The variance explained in their study was substantially higher than in our own findings, which may be due to differences in context (educational vs. organizational settings) or in how creativity was measured.

Another notable interaction emerged between gender and participation in creative activities inside school, indicating that the association between in‐school activities and creative thinking differed by gender. Although the main effect of gender showed an advantage for girls compared to boys, and the main effect of participation in creative activities inside school was negative, the interaction revealed that this effect was reversed across genders. Consistent with Milgram and Milgram (1976), who demonstrated that gender moderates the relation between the type of creative activity and creative thinking, our findings similarly indicated that gender moderated the association between participation in creative activities inside school and creative thinking scores, such that in‐school participation was beneficial for boys. One possible explanation is that participation in creative activities may be more socially stigmatized for 15‐year‐old boys; consequently, boys who choose to engage in these activities despite the stigma may represent a self‐selected group with higher creative thinking ability.

Despite the small effect sizes, these findings suggest that the benefits of extracurricular creative engagement may depend heavily on psychological and demographic traits as well as contextual support. This pattern is not that uncommon when investigating the effects of extracurricular activities on students' academic achievement and attitudes (Dumais, 2006). For example, Shulruf and colleagues (2007) reported similarly small effects, explaining up to 2% of the variance in students' academic achievement and their attitudes towards literacy and numeracy, and argued that participation in activities may exert only a modest influence on students' outcomes.

Taken together, these results highlight the importance of how creativity is defined and assessed in large‐scale testing. They also call for a deeper understanding of how various creative activities interact with psychological, demographic and contextual characteristics to support creativity.

## CONCLUSIONS AND FUTURE DIRECTIONS

Our analysis revealed significant population‐level patterns on the relation between creative activities participation and creative thinking performance meriting scholarly attention, though several limitations constrain interpretation. First, the large‐scale assessment design—with its planned missingness and plausible values estimation via conditioning models—precludes individual‐level inferences or specific classroom‐level pedagogical recommendations. We can only examine relations embedded within PISA's analytical framework, recovering patterns that reflect its underlying measurement assumptions. Because plausible values were imputed using background questionnaire data, most likely including students' participation in creative activities inside and outside of school, there is a model‐implied dependency between those imputation variables and the creative thinking outcome. Consequently, the relations estimated in the present analyses were, by definition, embedded within the conditioning model used to impute the plausible values. Nevertheless, this does not imply that secondary analyses lack value. Because the full specification of the conditioning model, including the complete set of included variables and the strength of the relations between them, is not transparently published, secondary analyses can recover meaningful associations and provide informative insights at the population level. In addition, this secondary analysis focused on a small number of theoretically chosen predictors, thereby honing in on the effects that were most relevant to our research questions. For further discussion of the assumptions and theoretical implications associated with secondary analysis of plausible values, see Dumas et al. (2026).

And second, while we employed appropriate techniques for clustered data analysis, an ideal approach would implement multilevel modelling to fully account for the nested structure of the data (students within schools within countries). Such analysis would require computational resources beyond typical laptop capabilities, suggesting the need for high‐performance computing solutions in future research.

Nevertheless, these findings compel us to re‐examine fundamental assumptions about creativity development in education. Several critical questions emerge: Do current creative activity formats effectively foster creativity, or do they require substantive redesign? Have extracurricular activities become overly institutionalized, adopting the rigid structures of formal schooling at the expense of their creative benefits? Alternatively, might some activities provide excessive autonomy, lacking the meaningful constraints and structure that research shows are equally vital for creative development (Beghetto, 2017)? Could it be that highly creative teenagers are simply not self‐selecting into the kinds of extracurricular activities that adults view as creative? To what extent do gender and activity type interact in predicting creative thinking, and do certain activities differentially benefit boys or girls? How might we better align both creativity assessments and creative activities with authentic creative processes? We see the current study as a key motivator for future research that asks and empirically answers these critical questions.

While unable to guide individual interventions, these macro‐level insights challenge prevailing assumptions and highlight the need for more nuanced measures capturing both convergent and divergent thinking in creativity, research on optimal creative activities structure and duration and investigations of how psychological and contextual factors mediate or moderate creative thinking performance measures' outcomes. We believe that future work examining the influence of different types of activities separately (e. g., art classes, creative writing, debate club), rather than collapsing them into two composite scores for in‐school and out‐of‐school activities, and accounting for the intensity, breadth and duration of participation could ultimately clarify the relation between creative activities and thinking. Even the OECD report (2024a) suggested that participation specifically in art classes was positively associated with students' creative performance in the visual expression domain of the PISA creative thinking test, although they did not provide the disaggregated creative thinking scores needed to empirically investigate this claim.

Ultimately, these findings serve their most vital purpose: prompting rigorous reflection on how extracurricular activities might truly cultivate creativity for all students, rather than being beneficial just for some.

## AUTHOR CONTRIBUTIONS

**Sofiia Kagan:** Conceptualization; methodology; formal analysis; writing – original draft; writing – review and editing. **Denis Dumas:** Supervision; writing – review and editing. **Yoojoong Kim:** Writing – review and editing.

## FUNDING INFORMATION

No funding was received for this study.

## CONFLICT OF INTEREST STATEMENT

The authors declare no conflicts of interest.

## ETHICS STATEMENT

This work follows all ethical guidelines of the American Psychological Association.

## Supporting information


Data S1:

## Data Availability

The data that support the findings of this study are openly available in PISA 2022 Database at https://www.oecd.org/en/data/datasets/pisa‐2022‐database.html.
