# Supplementary material for: Revisiting educational assumptions: The surprising negative link between creative extracurricular activities and creative thinking in PISA 2022
Source: Br J Educ Psychol. 2026 Feb 25;96(3):1080–101. doi: 10.1111/bjep.70066 (PMC13441010; doi:10.1111/bjep.70066)
Supplement: Supplementary file 1 [file BJEP-96-1080-s001.docx]

**Supplementary materials**

**Appendix A**

*Countries Included in the Analysis and Number of Students per Country*

| Albania | United Arab Emirates | Belgium | Bulgaria | Brazil | Bruney | Chile | Colombia | Czech Republic |
| --- | --- | --- | --- | --- | --- | --- | --- | --- |
| 6129 | 24600 | 8286 | 6197 | 10798 | 5576 | 6488 | 7804 | 8460 |
| Dominican Republic | Estonia | Finland | France | Greece | Croatia | Hungary | Indonesia | Iceland |
| 6868 | 6392 | 10239 | 6770 | 6403 | 6135 | 6198 | 13439 | 3360 |
| Italy | Jordan | Kazakhstan | Korea | Lithuania | Macao | Morocco | Mexico | North Macedonia |
| 10552 | 7799 | 19769 | 6454 | 7257 | 4384 | 6867 | 6288 | 6610 |
| Malta | Mongolia | Malaysia | Peru | Philippines | Poland | Portugal | Palestinian Authority | Qatar |
| 3127 | 6999 | 7069 | 6968 | 7193 | 6011 | 6793 | 7905 | 7676 |
| Azerbaijan | Ukrainian Regions | Romania | Saudi Arabia | Singapore | El Salvador | Serbia | Slovak Republic | Slovenia |
| 7720 | 3876 | 7364 | 6928 | 6606 | 6705 | 6413 | 5824 | 6721 |
| Chinese Taipei | Thailand | Uruguay | Uzbekistan |  |  |  |  |  |
| 5857 | 8495 | 6618 | 7293 |  |  |  |  |  |

**Appendix B**

*Number of Participants in Creative Activities Inside School by Country*

| Albania | United Arab Emirates | Belgium | Bulgaria | Brazil | Bruney | Chile | Colombia | Czech Republic |
| --- | --- | --- | --- | --- | --- | --- | --- | --- |
| 3398 | 18222 | 5142 | 4032 | 6516 | 4043 | 4400 | 5697 | 6185 |
| Dominican Republic | Estonia | Finland | France | Greece | Croatia | Hungary | Indonesia | Iceland |
| 2804 | 5588 | 7346 | 4520 | 3933 | 4656 | 4487 | 11807 | 2320 |
| Italy | Jordan | Kazakhstan | Korea | Lithuania | Macao | Morocco | Mexico | North Macedonia |
| 7431 | 4963 | 17839 | 6061 | 6039 | 4237 | 3845 | 4628 | 5026 |
| Malta | Mongolia | Malaysia | Peru | Philippines | Poland | Portugal | Palestinian Authority | Qatar |
| 2291 | 5513 | 5796 | 4136 | 6144 | 4426 | 4949 | 5789 | 4637 |
| Azerbaijan | Ukrainian Regions | Romania | Saudi Arabia | Singapore | El Salvador | Serbia | Slovak Republic | Slovenia |
| 2654 | 2609 | 5582 | 4968 | 5859 | 3814 | 4732 | 4284 | 5249 |
| Chinese Taipei | Thailand | Uruguay | Uzbekistan |  |  |  |  |  |
| 5353 | 7981 | 3535 | 4168 |  |  |  |  |  |

**Appendix C**

*Number of Participants in Creative Activities Outside School by Country*

| Albania | United Arab Emirates | Belgium | Bulgaria | Brazil | Bruney | Chile | Colombia | Czech Republic |
| --- | --- | --- | --- | --- | --- | --- | --- | --- |
| 3146 | 17483 | 5456 | 3841 | 5996 | 3696 | 4127 | 5343 | 6577 |
| Dominican Republic | Estonia | Finland | France | Greece | Croatia | Hungary | Indonesia | Iceland |
| 2619 | 5504 | 7155 | 4414 | 4498 | 4595 | 4473 | 10933 | 2222 |
| Italy | Jordan | Kazakhstan | Korea | Lithuania | Macao | Morocco | Mexico | North Macedonia |
| 7602 | 4629 | 17249 | 5883 | 5913 | 3664 | 3505 | 4448 | 4244 |
| Malta | Mongolia | Malaysia | Peru | Philippines | Poland | Portugal | Palestinian Authority | Qatar |
| 2308 | 4788 | 5497 | 3811 | 5809 | 4696 | 4865 | 5286 | 4377 |
| Azerbaijan | Ukrainian Regions | Romania | Saudi Arabia | Singapore | El Salvador | Serbia | Slovak Republic | Slovenia |
| 2368 | 2555 | 4803 | 4803 | 5586 | 3518 | 4550 | 4225 | 5216 |
| Chinese Taipei | Thailand | Uruguay | Uzbekistan |  |  |  |  |  |
| 3770 | 7654 | 3294 | 4022 |  |  |  |  |  |
